# Differential Effects of Tango Versus Dance for PD in Parkinson Disease

**DOI:** 10.3389/fnagi.2015.00239

**Published:** 2015-12-21

**Authors:** Marie E. McNeely, Marina M. Mai, Ryan P. Duncan, Gammon M. Earhart

**Affiliations:** ^1^Program in Physical Therapy, Washington University in St. Louis School of Medicine, St. Louis, MO, USA; ^2^Department of Neurology, Washington University in St. Louis School of Medicine, St. Louis, MO, USA; ^3^Department of Anthropology, Washington University in St. Louis School of Medicine, St. Louis, MO, USA; ^4^Department of Anatomy and Neurobiology, Washington University in St. Louis School of Medicine, St. Louis, MO, USA

**Keywords:** Parkinson disease, dance, tango, gait, balance, mobility

## Abstract

Over half of the general population does not achieve recommended daily levels of physical activity, and activity levels in people with Parkinson disease (PD) are lower than in healthy older adults. Dance can serve as an adjunct to traditional treatments to improve gait, balance, and quality of life in people with PD. This study directly compares a tango dance intervention and a dance intervention based on the Dance for PD model, which integrates multiple dance styles. Eleven people with PD participated in a community-based mixed styles dance intervention called Dance for Parkinson’s (D4PD). Participants in the D4PD group were matched to participants in an ongoing community-based exercise study who participated in tango dance. The groups received 12 weeks of intervention, attending 1-h group classes twice a week. Participants were evaluated off anti-PD medication before and after intervention. Measures of balance, repeated sit-to-stand performance and endurance (mini-balance evaluation systems test, four square step test, five times sit to stand, 6-min walk time) improved from pre to post similarly in both groups. Motor sign severity (movement disorders society unified Parkinson disease rating scale motor subsection) and functional mobility (timed up and go) improved in the tango group and worsened in the D4PD group. Gait velocity was not affected by either intervention. Direct comparisons of different interventions are ­critical for developing optimal exercise interventions designed to specifically target motor impairments in PD. Tango dance interventions may preferentially improve mobility and motor signs in people with PD, compared to D4PD.

## Introduction

Parkinson disease (PD) is the second most common neurodegenerative disorder, affecting 1–1.5 million Americans. PD is characterized by numerous non-motor and motor features, including gait and balance dysfunction. Gait dysfunction is of particular concern in PD, as it is most often the first area of difficulty reported by people with PD and is thought to represent the leading edge of disability (Shulman et al., 2008). As a result, the emergence of gait difficulty is considered a red flag (Morris et al., 2001, 2010). These gait difficulties have been noted in PD not only for forward walking, but also for backward walking, in which gait speed and stride length are even more reduced relative to healthy controls (Hackney and Earhart, 2009a, 2010a). While pharmacological and surgical approaches to the management of PD can help to partially alleviate some gait problems, they do not completely address the issue, indicating a need for additional and complementary approaches to the treatment of gait in PD.

Numerous studies have demonstrated the effectiveness of exercise as a complementary treatment for improving gait function in PD. Among the exercise approaches known to improve walking, dance has recently emerged as a promising therapy for improving gait, balance, and mobility while also reducing disease severity and improving quality of life [for reviews, see Earhart (2009), Herman et al. (2009), and Mehrholz et al. (2010)]. Tango dance specifically has been shown in several studies to improve a multitude of motor and non-motor features in people with PD (Hackney et al., 2007; Hackney and Earhart, 2009b,c,d, 2010b,c; Duncan and Earhart, 2012, 2014; Foster et al., 2013; McKee and Hackney, 2013; Hackney and McKee, 2014; Blandy et al., 2015), but other types of dance have been evaluated as well. Interventions using the trademarked Dance for PD class model (Brooklyn Parkinson Group MMDG, 2010–2015) in particular have demonstrated positive effects on motor control and quality of life in preliminary, uncontrolled studies (Westheimer, 2008; Heiberger et al., 2011; Westheimer et al., 2015). While previous dance studies have helped identify possible intervention approaches to improve gait and mobility in people with PD, many studies are conducted without adequate control groups. Furthermore, there are few direct comparisons between different dance interventions to determine which may provide the most benefit for particular deficits in PD. Our study is the first to compare the effects of tango and the Dance for PD model on motor function, gait, balance, and quality of life. This study provided the opportunity to compare potential benefits of a dance intervention modeled off of the Dance for PD program to a tango dancing class, which has been more commonly studied in people with PD. We hypothesized that tango dance classes may provide greater benefits in gait, balance, and mobility compared to the Dance for Parkinson’s (D4PD) class because the Dance for PD model includes a substantial amount of time spent seated, whereas participants in the tango class spend more time standing and stepping. We expected both classes to provide equivalent improvements in quality of life.

This direct comparison of two different dance interventions administered with similar intervention parameters provides a valuable contribution to current knowledge on whether certain dance programs target particular impairments in people with PD.

## Materials and Methods

### Participants

Participants with idiopathic PD were recruited for both groups from the Washington University School of Medicine Movement Disorders Center. All participants were screened to meet the following inclusion criteria: a neurological diagnosis of PD (diagnostic criteria included those used for clinically defined “definite PD,” as previously described by Racette et al. (1999) based on established criteria (Calne, 1992; Hughes et al., 1992)); clear benefit from levodopa; able to stand independently for at least 30 min; no evidence of dementia (MMSE ≥ 26); no serious medical problem (aside from PD); no evidence of abnormality on brain imaging (previously done for clinical evaluations – not part of this research); no history or evidence of other neurological deficit, such as previous stroke or muscle disease; no deep brain stimulation; and no other recent surgeries or injuries affecting movement. The study was approved by the Institutional Review Board of Washington University in St. Louis Medical School. All participants gave informed verbal and written consent prior to their participation in the study.

Eleven participants with PD were enrolled in the D4PD intervention. Eight participants (four women and four men) completed the classes and both assessments (Table 1). Reasons for dropouts included a leg injury unrelated to the class, scheduling conflicts, and increased family member care responsibilities. The eight participants who completed the intervention were matched to a subset of participants with PD who participated in tango classes as part of a separate ongoing study (Table 1) (Earhart et al., 2015).

**Table 1:** **Demographic characteristics**.

	D4PD	Tango
Total (*n*)	8	8
Males/females	4/4	4/4
Age (years)	68.25 ± 10.90	67.66 ± 8.62
MMSE	28.50 ± 1.41	28.63 ± 1.19
Disease duration (years)	10.06 ± 4.14	5.38 ± 4.83
MDS-UPDRS-III	31.00 ± 17.01	32.63 ± 6.86
H&Y stage	2.25 ± 0.27	2.13 ± 0.58

*Values are M ± SD. In D4PD, H&Y scores were 2 for four participants and 2.5 for four participants. In Tango, H&Y scores were 1 for one participant, 2 for four participants, 2.5 for two participants, and 3 for one participant*.

### Dance Interventions

Participants participated in 12 weeks of dance, meeting two times per week for 1 h each session. Attendance at 80% of the classes was required for inclusion in the study. Make-up classes were offered for participants who were on the borderline for attendance. Both the tango and D4PD groups exercised in the same community-based group setting on the Washington University School of Medicine campus to ensure that participants had similar experiences with respect to social interactions associated with participating in a group exercise class.

#### D4PD Classes

An undergraduate dance student (MMM) from Washington University in St. Louis with 17 years of dance training in ballet and modern, including 5 years in a pre-professional youth company, led the D4PD classes. Volunteer students from the Performing Arts Department at Washington University assisted in the classes each week, dancing with the participants, demonstrating material, and providing individual support for anyone with balance problems. Instruction was tailored to the level of the class, but participants were reminded to modify exercises or difficult movements if necessary.

The D4PD class material was based on the Dance for PD model [a program created by dancers in the Mark Morris Dance Group in conjunction with the Brooklyn Parkinson’s Group (Brooklyn Parkinson Group MMDG, 2010–2015)]. The curriculum was drawn from the instructor’s training received at a certified Dance for PD introductory training workshop taught by founding teachers David Leventhal and Misty Owens, as well as observation of certified Dance for PD classes, the instructor’s experience leading 6 weeks of D4PD pilot classes in St. Louis, and online Dance for PD membership resources. The classes were accompanied by a playlist of songs ranging from classical piano and Broadway show-tunes to generational pop, such as the Beatles and Bee Gees, in accordance with suggestions at the training, established classes, and online member resources.

Classes began with a 30-min seated warm-up in chairs. Seated movements focused on arm movements, foot and ankle movements, articulation of the spine and head, and facial expressions. Movements ranged in quality, sharp to continuous, and in speed. A cognitive activity challenging memory, rhythm, or sequence order also took place. This was followed by 5 min at the “barre,” which for our class were the handrails of treadmills. The barre combinations concentrated on bigger movements, making shapes, extending through lines made by the body, bending of the knees, and testing/finding balance. The last 25 min were devoted to moving across the floor, integrating the whole body in coordinated movements. This portion of the class was often a mixture of choreographed sequences, improvisation, theatrical interpretation, and group dancing. Though the classes were based in ballet and modern dance, aspects of choreographic repertory, theater dance, jazz, tap, square dancing, Irish dancing, salsa, and flamenco were also incorporated.

Teaching methods included verbal instruction, imagery, visualization, repetition, cognitive activities, and variations on movement, including direction, speed, quality of movement, and sequence. Each class included elements of improvisation and creativity, such as creating their own movement sequences, shaping movements of others as if they were “clay statues,” mirroring each other’s movement, passing a gesture around the circle, making an artistic choice, and building a group dance to music.

#### Tango Classes

The Tango comparison group classes were conducted as part of an ongoing study and were modeled on those of our previous studies (Hackney and Earhart, 2009b,c; Duncan and Earhart, 2012). The instructors for the tango classes were two graduate students who were experienced tango dancers with prior experience teaching tango. The two partnered with each other for demonstration purposes during the class and followed a curriculum they developed based on prior tango studies (Hackney, 2010; Duncan and Earhart, 2012). Partners were individuals without PD and included spouses and caregivers of those with PD as well as undergraduate volunteers from Washington University in St. Louis. The undergraduate volunteers assisted in the classes each week, dancing with the participants, demonstrating material, and providing individual support for anyone with balance problems. Instruction was tailored to the level of the class, but participants were reminded to modify exercises or difficult movements if necessary.

Music selections were standardized and included songs with and without lyrics that had a clear beat and reasonable tempo. Tango classes began with a brief warm-up that focused on range of motion of all joints, trunk rotations, and weight shifts. This was followed by 45 min of instruction and partnered tango dancing. Participants danced both leading and following roles and changed partners to ensure that everyone spent time moving forward and backward and got experience dancing with different partners. In the leading role, participants practiced self-directed, internally generated movements, while the following role involved recognizing and responding to external cues from their dance partner (Hackney, 2010). Traditional tango steps were often modified to accommodate the abilities and balance limitations of the participants (Hackney, 2010). Emphasis was placed on practicing weight shifts, walking backward, proper posture, rhythmic training, and moving with a partner. There was a brief cool down period at the end of each class. The tango classes were progressive in nature with new steps and sequences being added and integrated with known steps over the course of the intervention.

### Assessments

Participants were tested before and after the 12-week dance intervention in the practically defined off medication state after withholding medication overnight (i.e., at least 12 h since previous medication administration). Baseline evaluations were conducted within 1 month prior to the start of the dance classes, and post-test evaluations were conducted within 1 month of completion of the dance classes.

The pre-intervention evaluation (PRE) included the following assessments: mini mental status exam (MMSE), movement disorders society unified Parkinson disease rating scale motor subsection (MDS-UPDRS-III), mini-balance evaluation systems test (Mini-BESTest), 6-min walk, five times sit to stand, four square step test, and Parkinson’s disease questionnaire-39 (PDQ-39). In addition, gait was assessed during forward preferred speed, forward fast as possible, backward, and dual task walking using a 4.8m GAITRite instrumented walkway system (CIR Systems, Havertown, PA, USA). Dual task walking was done at the preferred pace while the participant completed a phonemic listing tasks (naming words that begin with H, L, and T for each of the three trials, respectively). Counts of correct words and errors were recorded. We collected three trials of each gait condition, and the order of the conditions was randomized for each participant. Balance was assessed by a trained physical therapist using the Mini-BESTest (Franchignoni et al., 2010). Times to complete the timed up and go (TUG) and dual task timed up and go (DT-TUG) were extracted from the Mini-BESTest to measure functional mobility with and without a dual task. The post-intervention evaluation (POST) was conducted upon completion of the 12 weeks of classes, and exactly mirrored the PRE.

### Analysis

Mixed model ANOVAs were run in SPSS Version 22 with group (D4PD and tango) and time (pre and post) as factors to determine whether and how different forms of dance impact normalized gait velocity for forward and backward walking. For each gait condition, three trials were averaged for each participant. Normalized gait velocity, MDS-UPDRS-III, and Mini-BESTest scores were our primary variables of interest to evaluate changes in gait, motor sign severity, and balance. Data that were not continuous (Mini-BESTest scores, UPDRS-III scores, and PDQ-39 Index scores) underwent aligned rank-transformation using the ARTool (Wobbrock et al., 2011) prior to analysis with mixed model ANOVAs in SPSS. The aligned rank-transform method addresses the issue of increased type one errors for interactions that occur with standard rank-transform procedures (Wobbrock et al., 2011). Mixed model ANOVAs were also performed for secondary variables of interest (TUG, DT-TUG, 6-min walk, four square step test, five times sit to stand, and PDQ-39). Post hoc paired analyses were conducted as appropriate.

## Results

Average attendance at each class was 7.46 ± 1.10 participants. Groups did not differ significantly at baseline in age, MMSE, disease duration, or MDS-UPDRS-III scores (*p* > 0.05).

### Motor Sign Severity and Quality of Life

**Figure 1:**
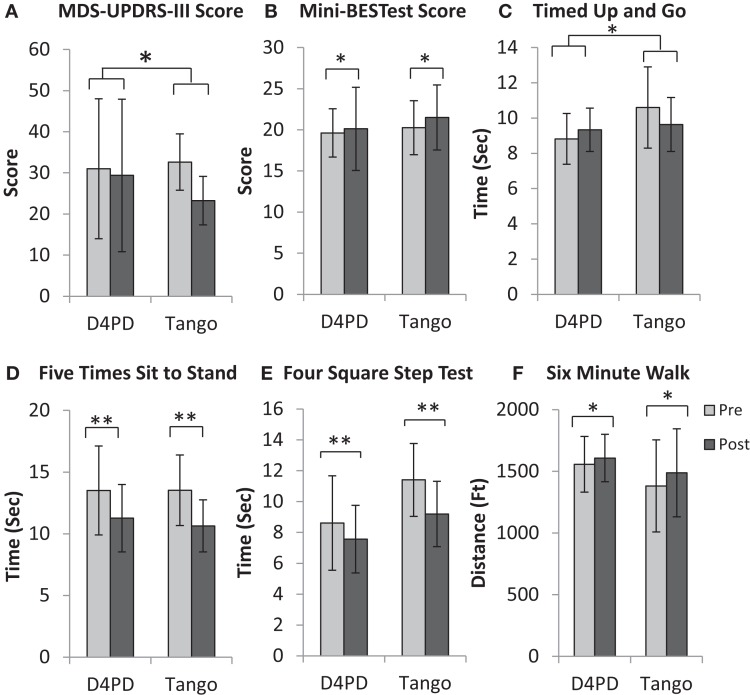
**Movement disorders society unified Parkinson disease rating scale motor subsection [MDS-UPDRS-III, (A)], mini-balance evaluation systems test [Mini-BESTest, (B)], timed up and go (C), five times sit to stand (D), four square step test (E), and 6-min walk (F) for participants in the Dance for Parkinson’s (D4PD) and tango interventions**. Significant group × time interaction for MDS-UPDRS-III score and timed up and go. Significant main effect of time for Mini-BESTest score, five times sit to stand, four square step test, and 6-min walk, *p ≤ 0.05, **p ≤ 0.01. Values are M ± SD.

For MDS-UPDRS-III, there was a significant time by group interaction [*F*(1,14) = 8.607, *p* = 0.011] and a significant effect of time [*F*(1,14) = 19.217, *p* = 0.001]. There was no group effect on MDS-UPDRS-III [*F*(1,14) = 0.003, *p* = 0.958]. On average, UPDRS-III scores improved (decreased) more from pre- to post-test in the tango group (Figure 1A; Table 2). There were no significant interaction, time, or group effects, respectively for the PDQ-39 Index scores [*F*(1,14) = 0.787, *p* = 0.390; *F*(1,14) = 0.151, *p* = 0.703; *F*(1,14) = 0.782, *p* = 0.391; Table 2].

**Table 2:** **Pre- and Post-test measurements for all variables of interest**.

	D4PD		Tango	
	Pre	Post	Pre	Post
MDS-UPDRS-III	31.00 ± 17.01	29.38 ± 18.55	32.63 ± 6.86	23.25 ± 5.90
PDQ-39	126.51 ± 110.63	131.09 ± 115.02	135.05 ± 57.14	107.55 ± 55.04
Mini-BESTest	19.63 ± 2.92	20.13 ± 5.06	20.25 ± 3.28	21.50 ± 3.96
TUG (s)	8.82 ± 1.44	9.33 ± 1.23	10.60 ± 2.30	9.63 ± 1.53
DT-TUG (s)	10.93 ± 1.85	11.57 ± 1.44	12.04 ± 3.15	12.48 ± 2.51
Five × sit to stand (s)	13.51 ± 3.60	11.26 ± 2.73	13.53 ± 2.86	10.64 ± 2.11
Four square step (s)	8.61 ± 3.06	7.57 ± 2.19	11.41 ± 2.37	9.19 ± 2.11
6-min walk (ft)	1556.75 ± 225.99	1607.00 ± 192.18	1381.88 ± 372.98	1488.00 ± 356.10
Normalized forward velocity	1.53 ± 0.15	1.51 ± 0.27	1.39 ± 0.22	1.36 ± 0.25
Normalized dual velocity	1.12 ± 0.14	1.18 ± 0.11	1.17 ± 0.25	1.19 ± 0.33
Normalized fast velocity	2.07 ± 0.24	2.16 ± 0.26	1.85 ± 0.28	1.92 ± 0.41
Normalized backward velocity	1.01 ± 0.14	1.00 ± 0.21	0.81 ± 0.22	0.90 ± 0.27

*Values are M ± SD. Gait velocities (centimeter per second) are normalized to participants’ leg lengths (centimeter)*.

### Balance and Mobility

Mini-BESTest results indicated that there was no significant interaction effect [*F*(1,14) = 0.124, *p* = 0.730], but a significant effect of time [*F*(1,14) = 5.167, *p* = 0.039]. There were no significant group effects [*F*(1,14) = 0.002, *p* = 0.968]. The Mini-BESTest scores increased (improved) from pre- to post-test (Figure 1B; Table 2).

For TUG, there was a significant group by time interaction [*F*(1,14) = 5.413, *p* = 0.036], but no main effects of time [*F*(1,14) = 0.510, *p* = 0.487] or group [*F*(1,14) = 1.802, *p* = 0.201]. On average, TUG times increased (worsened) from pre- to post-test in D4PD and decreased (improved) from pre- to post-test in Tango (Figure 1C; Table 2). By contrast, there were no interaction [*F*(1,14) = 0.068, *p* = 0.799], time [*F*(1,14) = 1.960, *p* = 0.183], or group [*F*(1,14) = 0.841, *p* = 0.375] effects for DT-TUG (Table 2).

Five times sit to stand demonstrated no significant interaction effect [*F*(1,14) = 0.191, *p* = 0.668], but there was a significant time effect [*F*(1,14) = 12.016, *p* = 0.004]. There was no group effect [*F*(1,14) = 0.059, *p* = 0.811]. The five times sit-to-stand test time decreased (improved) from pre- to post-test (Figure 1D; Table 2).

For four square step test, there was no interaction effect [*F*(1,14) = 1.635, *p* = 0.222], but there was a significant effect of time [*F*(1,14) = 12.661, *p* = 0.003]. There was no group effect [*F*(1,14) = 3.738, *p* = 0.074]. The four square step test time decreased (improved) from pre- to post-test (Figure 1E; Table 2).

On the 6-min walk test, there was no significant interaction [*F*(1,14) = 0.751, *p* = 0.401], but there was a significant effect of time [*F*(1,14) = 5.884, *p* = 0.029]. There was no group effect [*F*(1,14) = 1.024, *p* = 0.329]. Six-minute walk test distance increased (improved) from pre- to post-test (Figure 1F; Table 2).

### Gait Analysis

Forward preferred pace gait data were not available for one participant in the D4PD group. There were no group by time interactions for normalized velocity in forward [*F*(1,13) = 0.012, *p* = 0.913], dual task [*F*(1,14) = 0.250, *p* = 0.625], fast [*F*(1,14) = 0.071, *p* = 0.794], or backward [*F*(1,14) = 0.999, *p* = 0.335] gait conditions. There were also no significant time or group effects, respectively, for normalized velocity in forward [*F*(1,13) = 0.593, *p* = 0.455, *F*(1,13) = 2.167, *p* = 0.165], dual task [*F*(1,14) = 1.107, *p* = 0.311, *F*(1,14) = 0.091, *p* = 0.768], fast [*F*(1,14) = 2.966, *p* = 0.107, *F*(1,14) = 2.580, *p* = 0.131], or backward [*F*(1,14) = 0.690, *p* = 0.420, *F*(1,14) = 2.546, *p* = 0.133] gait conditions (Table 2).

There were no significant differences in the number of correct, error, and repeat responses in the cognitive portion of the dual task between groups or across time (*p* > 0.05), though there was a trend toward a group by time interaction in the number of correct responses [*F*(1,14) = 3.618, *p* = 0.078] where performance improved on average in the tango group and worsened in the D4PD group from pre- to post-test.

## Discussion

To our knowledge, this study is the first to directly compare the effects of a dance intervention based on the Dance for PD program with a comparison intervention. This is also one of the few studies in PD that directly compares the effects of two different exercise interventions on movement and quality of life outcomes in this population. Overall, both tango and D4PD dance groups demonstrated improvements in measures of balance and mobility. Quality of life and gait velocity during a variety of gait tasks did not improve with either intervention. The tango intervention was more effective than D4PD for improving outcomes related to motor sign severity and functional mobility.

### Motor Sign Severity and Quality of Life

In the present study, PD motor sign severity (MDS-UPDRS-III scores) improved more in the tango group from pre- to post-test. Previous uncontrolled preliminary studies of participation in one session or a 20-session dance interventions using the Dance for PD program found significant improvements in UPDRS-III total scores with 10.4–34.6% improvements on average (3.0–8.2 points) (Heiberger et al., 2011; Westheimer et al., 2015). Our participants were similar in age and motor sign severity to the previous study, but we observed only a 5.2% change on average (1.6 points), which would likely not be clinically meaningful. Differences in our results may be attributed to differences in the intervention administration (e.g., different instructors) or parameters (e.g., 1 h compared to 1 h and 15 min).

In previous tango intervention studies, participants improved UPDRS-III scores more than no-intervention (Hackney and Earhart, 2009b; Duncan and Earhart, 2012, 2014) and education-only controls (McKee and Hackney, 2013), but improvements were similar in a tango and an adapted strength/flexibility exercise control group (Hackney et al., 2007). MDS-UPDRS-III scores improved in our tango group by 28.7% on average (9.4 points). This is likely a clinically meaningful change.

Looking at our data for individual participants, 4 of the 8 in D4PD improved, 2 did not change, and 2 were worse at post-test, compared to baseline. In the tango group, scores for each of the participants improved. Anecdotally, there were no apparent differences in age, sex, freezing status, baseline MDS-UPDRS-III scores, or disease duration for those who improved and those who did not. It is possible that greater and more consistent improvements in the tango group may have occurred in our study because the tango group spent a larger proportion of class time practicing PD-specific impairments assessed by the MDS-UPDRS-III, such as stepping, posture, and rhythmic movements, compared to the D4PD group.

Neither our study nor previous studies examining Dance for PD programs observed significant changes in quality of life using the PDQ-39 summary index scores (Westheimer et al., 2015). Results have been mixed in the literature for improvements in quality of life (measured with the PDQ-39) with tango interventions, with one study reporting greater improvements with tango compared to ballroom, tai chi, and controls (Hackney and Earhart, 2009d), but other studies reported similar changes in tango and control groups (McKee and Hackney, 2013; Rios Romenets et al., 2015). Scores on the PDQ-39 were variable, and there was particularly high variability in the D4PD group.

Despite lack of differences in physical, quality of life, and mood-related measures, participants in D4PD classes have reported perceived improvements in physical, social, and emotional states in a self-report interview form, as well as on items of the quality of life scale from the Oregon Health and Sciences University, such as participation in recreational activities, socializing, and their physical heath (Westheimer, 2008; Heiberger et al., 2011). Qualitative improvements have also been reported in PD with tango interventions (Hackney and Earhart, 2009b, 2010b). Participants in both D4PD (Westheimer et al., 2015) and tango interventions have also expressed interest in continued participation in the class (Hackney and Earhart, 2009b, 2010b; Duncan and Earhart, 2012). It is possible that measurements such as the PDQ-39 may not be capturing some of the benefits people with PD may receive by participating in dance classes (Westheimer et al., 2015).

### Balance and Mobility

Balance performance, measured using the Mini-BESTest, improved in both dance intervention groups. It should be noted that the improvements were small (0.5 points and 1.25 points for D4PD and tango, respectively) and may not be clinically meaningful. A previous study reports the minimum detectable change for the Mini-BESTest in patients with balance disorders was 3.5 points (Godi et al., 2013). Improvements in balance with tango interventions have been demonstrated in PD using various clinical balance scales (Mini-BESTest, Berg Balance Scale, and Fullerton Advanced Balance Scale) (Hackney et al., 2007; Hackney and Earhart, 2009b, 2010b; Duncan and Earhart, 2012, 2014; McKee and Hackney, 2013). However, our improvements in the D4PD group conflict with a previous study that did not demonstrate significant improvements in balance, measured using the Berg Balance Scale, with a Dance for PD intervention (Westheimer et al., 2015). Discrepant results may be due to the differences in balance scales used since the mini-BESTest is less susceptible to ceiling effects in PD, compared to the Berg Balance Scale (Schlenstedt et al., 2015).

Participants in the tango group in the present study showed an improvement in TUG scores with intervention, compared to members in the D4PD group (where performance declined on average). In previous tango studies, TUG performance did not change significantly with intervention (Hackney et al., 2007; Hackney and Earhart, 2009b, 2010b; McKee and Hackney, 2013; Duncan and Earhart, 2014), though non-significant improvements have been reported (Hackney et al., 2007; Hackney and Earhart, 2009b; McKee and Hackney, 2013). Furthermore, TUG performance did not change significantly in a previous study after one session of a Dance for PD-based dance class (Heiberger et al., 2011), but participants in our D4PD group experienced a slight worsening of TUG performance over the course of the study. The subtle worsening seen in our study may potentially reflect worsening of PD symptoms over the course of 3 months that would not have been detected after just one session in the previous study. However, the average changes observed in our study were small (0.5 s worse in D4PD and 1.0 s improvement in tango), and minimal detectable change for TUG in PD is reported to be 3.5 s (Huang et al., 2011).

Looking at data for individual participants, four people in the D4PD group improved TUG times and performance worsened for 4 D4PD participants. In the tango group, five participants improved and three worsened over the course of the study. There were again no apparent differences in age, sex, freezing status, baseline MDS-UPDRS-III scores, or disease duration for those who improved and those who did not. Though results in both groups were variable, it is possible that greater improvements may have occurred in the tango group because the tango group spent a larger proportion of class time standing, stepping, and turning compared to the D4PD group, and these are all important components of TUG performance.

Other measures of balance and mobility (five times sit to stand, four square step test, and 6-min walk) all demonstrated significant main effects of time, with similar improvements in both dance intervention groups. Six-minute walk test performance has improved consistently in previous studies with tango (Hackney and Earhart, 2009b, 2010b; Duncan and Earhart, 2012, 2014) and waltz/foxtrot dance interventions (Hackney and Earhart, 2009b). Five times sit to stand improved with Turkish Folkloristic dancing in PD (Eyigor et al., 2009), but has not previously been evaluated in tango or Dance for PD-based interventions. The four square step test has been shown to improve following cycling in people with PD (Uygur et al., 2015), but this is the first study to evaluate the effects of dance interventions on this measure in PD. Participating in the weekly hour-long exercise dance classes may have improved physical endurance in both groups, resulting in improved 6-min walk distance, despite the fact that a portion of the D4PD class was conducted in chairs. Practicing aspects of movement, such as weight shifting and dynamic balance, may have helped participants in both classes perform the five times sit to stand test more quickly. These skills, as well as practice of relatively complex step sequences in both classes, may have contributed to improvements in the four square step test in both groups.

### Gait

We anticipated that participants in the tango group would experience greater improvements in walking, particularly backward walking, because these activities would be practiced more during the tango class than the D4PD class. Neither participants in the tango nor the D4PD groups exhibited significant changes in gait velocity in any gait condition tested. Dance interventions in PD have had inconsistent effects on gait, with some reports of improvement and others reporting no change (Hackney et al., 2007; Hackney and Earhart, 2009b, 2010b; Duncan and Earhart, 2012, 2014). It is important to note that in the present study we included people with mild to moderate PD, and raw gait speeds for the participants in both dance groups tended to be in the mid-range of normal gait speeds for healthy adults of similar ages (Bohannon and Williams Andrews, 2011). It is possible that dance interventions may be able to provide larger benefits in gait speed for individuals with greater gait impairments, but are less effective for people who already walk at near-normal speeds.

### Adherence

A previous study using Dance for PD programs reported low attrition rates [12/14 (86%) enrolled completed] (Westheimer et al., 2015). By contrast, our attrition rate for the D4PD group was higher [8/11 (73%) completed]. Reasons provided for drop outs in our study were not related to the intervention (one for an unrelated leg injury and two for competing/other commitments).

### Limitations and Future Directions

Limitations of our present study include the small sample size for both dance groups. This study and all previous studies evaluating Dance for PD-based interventions have been small, with fewer than 15 participants per group (Heiberger et al., 2011; Westheimer et al., 2015). In addition, the present study included participants with mild-to-moderate disease severity. Studies focusing on participants of different levels of disease severity may allow instructors to provide more targeted content and clarify whether improvements with dance in motor sign severity, quality of life, balance, mobility, and gait differ across the course of the disease. Larger controlled studies evaluating Dance for PD and other types of dance interventions are necessary to determine whether certain dance intervention characteristics (i.e., dance style, skills practiced, activity levels, class duration, etc.) may be optimal for addressing certain deficits in people with PD.

Few dance studies in the PD literature directly compare different dance interventions. In order to provide recommendations for people with PD on which interventions may best address their symptoms and impairments, as well as promote adherence, it will be important to conduct additional studies with larger sample sizes, so various exercise interventions can be compared. Comparisons between literature studies are hampered by differences in study design, including intervention parameters used (class length, frequency, intervention duration), type of exercise/dance trained, and outcomes measured.

## Conclusion

Participation in a dance intervention similarly improved measures of balance and mobility in people with PD, regardless of whether participants were engaged in tango dancing or a class modeled on the Dance for PD program. However, the tango intervention provided greater improvements in measures of motor sign severity and functional mobility.

This study is the first to compare the effects of a Dance for PD-based intervention to a tango dance intervention in people with PD. We also used measures, including the five times sit to stand and the four square step test, that have not previously been used to evaluate outcomes for either type of dance intervention in PD. Future research is needed to better understand characteristics of dance classes that are optimal for improving function in people with PD and whether there are subsets of participants who may respond better to these interventions.

## Author Contributions

MMM provided substantial contributions to the design of this study, the development and delivery of the dance intervention, and data entry. RD contributed substantially to the study design and data acquisition during the pre-test and post-test evaluations. MEM contributed substantially to the study design, as well as data analysis and interpretation. GE provided substantial contributions to the study design and data interpretation. MMM and MEM drafted the manuscript. All authors contributed substantially to revising and critically reviewing the manuscript draft. Furthermore, all authors approved the final manuscript version and agree to be accountable for all aspects of the work.

## Conflict of Interest Statement

This research was conducted in the absence of any commercial or financial relationships that could be construed as a potential conflict of interest.

## Abbreviations

D4PD, dance for Parkinson’s dance class based on dance for PD model; MDS-UPDRS-III, movement disorders society unified Parkinson disease rating scale motor subsection; Mini-BESTest, mini-balance evaluation systems test; MMSE, mini mental state exam; PD, Parkinson disease; PDQ-39, Parkinson’s disease questionnaire-39; TUG, timed up and go.
